# Evaluation of root resorption after comprehensive orthodontic treatment using cone beam computed tomography (CBCT): a meta-analysis

**DOI:** 10.1186/s12903-018-0579-2

**Published:** 2018-06-27

**Authors:** Yaqi Deng, Yannan Sun, Tianmin Xu

**Affiliations:** Department of Orthodontics, Peking University School and Hospital of Stomatology, Beijing, 100081 People’s Republic of China

**Keywords:** Root resorption, Cone beam computed tomography, Orthodontics, Meta-analysis

## Abstract

**Background:**

Orthodontic treatment can result in root resorption (RR). Traditional two-dimensional (2D) data exhibit magnification, deformation and positioning problems. Cone beam computed tomography (CBCT) contains more accurate three-dimensional (3D) information. This study identified and qualified the extent and location of root resorption using cone beam computed tomography (CBCT) after comprehensive orthodontic treatment.

**Methods:**

Studies comparing the RR before and after comprehensive orthodontic treatment using CBCT were identified using electronic searches of databases, including Cochrane, PubMed, EMBASE, China National Knowledge Infrastructure (CNKI) and Web of Science, and manual searches in relevant journals and the reference lists of the included studies until Oct 25, 2017. The extraction of data and the risk of bias evaluation were conducted by two investigators independently. The methodological quality of the included studies was assessed using the methodological index for non-randomized studies (MINORS). Studies that reported the length and volume of teeth were used for quantitative analyses.

**Results:**

Twelve studies were included in the meta-analysis. The length of all teeth after intervention was significantly shorter than that before treatment (MD = 0.80, 95% CI 0.56, 1.03, *P* < 0.00001). The sequence of RR from heaviest to lightest was maxillary lateral incisors, maxillary central incisors, mandibular anterior teeth, and maxillary canines. Studies were divided into two subgroups based on the use of tooth extraction. Root shortening after treatment was observed in both groups, and extraction caused more root resorption than was observed in the non-extraction group.

**Conclusions:**

There were different degrees of root resorption after orthodontics, but it was clinically acceptable. Root resorption established in CBCT research was less serious and more accurate than that observed in the two-dimensional research. Current evidence suggests that root length and volume were reduced after orthodontic treatment. The order of the amount of RR was maxillary lateral incisors, maxillary central incisors and mandibular anterior teeth. Most of the articles were complicated by different confounding factors. Therefore, more high-quality clinical trials are needed to determine the risk factors of root resorption and optimal protocols for treatment and to draw more reliable conclusions.

## Background

External apical root resorption (EARR) is a reduction in root structure involving the apices, and it is a common phenomenon of orthodontic treatment in the modern world [1]. Most resorption is clinically insignificant, but severe root resorption threatens tooth longevity and causes tooth mobility or loss [2]. With improvements in orthodontic techniques and increased patient expectations, orthodontists need to be aware of EARR [3].

The prevalence of EARR is high, and the factors affecting it are complex and multiple, including internal and external factors. Internal factors are patient factors that include genetics, age at the start of treatment, gender, nutrition, root morphology, alveolar bone density, type of malocclusion, and so on [4–6]. External factors are primarily caused by orthodontic treatment, such as the type of appliance, treatment technique, continuous or intermittent force, force magnitude and direction, duration of the applied force, premolar extractions, tooth distance and root movement are risk factors for EARR [7–9]. The causes and mechanisms of resorption are not completely clear.

Different aspects of tooth resorption, including prevalence and degree, were investigated using conventional radiographs. Conventional radiographs include periapical film, panoramic radiograph, and lateral cephalometric images. Image distortion and magnification are common characteristics of panoramic radiography, also known as non-positioning radiographs, and this technique imprecisely measures cephalometric points [10, 11]. The disadvantages of this approach include confounded images caused by superimposed anatomic structures and a lack of right- and left-side information [12].

However, root resorption occurs 3-dimensionally, and 2D images cannot detect root resorption on lingual or buccal surfaces nor measure the volume of root loss. Therefore, quantification of treatment outcome using 2D images raised some criticism because of its reliability.

CBCT is an effective imaging method for the diagnosis of orthodontic root resorption using a 1: 1 ratio for reconstruction with no amplification error [13]. CBCT clearly shows the root structure, which results in more accurate qualitative judgment of orthodontic root resorption [14, 15]. CBCT images enhance cross-section research in three dimensions because the images may be observed at any angle using 3D reconstruction. Therefore, studies on RR using CBCT demonstrated improved accuracy and sensitivity in comparison with those using 2D data [16]. CBCT data contain equal image information of the right and left sides with no interference due to image overlap. Wang demonstrated that CBCT accurately measured tooth and root resorption volumes, and it was a more accurate and reliable 3D measuring method for EARR investigation [17]. Another significant advantage of CBCT in root resorption studies is that it could be used in vivo, compared with Micro CT.

Weltman et al. [4] and Roscoe et al. [18] systematically reviewed root resorption associated with orthodontic treatment based on 2D images, but they did not do quantitative synthesis. There are no systematic reviews of root resorption associated with orthodontic treatment based on CBCT data, which is a more accurate and scientific method [19]. Therefore, it is necessary to integrate the data and conclusions of these trials. The purpose of this article is to report the results of a rigorous systematic review of the scientific literature relating to EARR in patients with fixed orthodontic appliances using the most accurate imaging information, CBCT.

## Methods

This meta-analysis was performed in accordance with the guidelines of the Preferred Reporting Items for Systematic Reviews and Meta Analyses (PRISMA) checklist and PRISMA harm checklist items.

### Types of studies

Study design: Randomized and non-randomized controlled trials, clinical trials, and prospective and retrospective reports were included. Longitudinal studies that observed root changes at different time points of treatment (before and after orthodontic treatment) were included. Self-controlled studies were included. Case reports, case series studies, descriptive studies, opinion articles and reviews were not included.

### Types of participants

We included studies of orthodontic patients with no restrictions in the characteristics of occlusion, age or gender, and with available pre-and post-operative CBCT data. Patients with periodontitis were excluded. Pregnant patients and patients with systemic diseases, syndromes, pathologies, or history of root resorption were excluded.

### Types of interventions

For comprehensive orthodontic treatment, patients in permanent dentition with fixed appliances, such as brackets and bands were included. Patients with different wire techniques and orthognathic surgery patients with pre-operative orthodontics with extraction treatment (bicuspid extraction on the upper and/or lower arch) or non-extraction were also included. Patients with local orthodontic treatment or stage treatment were excluded.

### Types of outcome measures

Primary outcomes: Root resorption was evaluated using CBCT after orthodontic treatment. The primary outcomes were tooth/root length and tooth/root volume.

The PICOS format and null hypothesis are shown in Table 1.

**Table 1:** PICOS format and null hypothesis

PICOS format	
Population	Patients with orthodontics
Intervention	Comprehensive orthodontics; not local orthodontics
Comparison	Before and after treatment
Outcome	Root resorption evaluated as tooth/root length and volume assessed using radiographic imaging CBCT
Null hypotheses	There is no difference in the incidence and severity of root resorption before and after comprehensive orthodontic treatment.

#### Search methods for study identification

For the identification of studies to include or consider for this review, we developed detailed search strategies for each database searched. These strategies were based on the search strategy developed for MEDLINE but revised appropriately for each database. We searched the following databases: the CNKI database, the Cochrane Library, Web of Science, PubMed and EMBASE (to October 2017). We used no language or date restrictions in the searches of the electronic databases. The key words used to screen the databases are shown in Table 2. Citations of the remaining studies were examined to identify publications not located in the MEDLINE database. We contacted the authors of randomized controlled trials to identify any unpublished trials.

**Table 2:** Search results

Data base	Search strategy	Numbers
CNKI	Subject = root resorption AND Subject = orthodontic AND Subject = CBCT (accurate match)	103
Cochrane	Root resorption: ti, ab kw and Orthodontics: ti, ab, kw and cone beam computed tomography: ti, ab, kw	8
Web of Science	TS = ((root resorption) AND orthodontics AND (CBCT OR (Cone Beam Computed Tomography)))	34
PubMed	(Cone Beam Computed Tomography))) (root resorption) AND (root resorption) AND (orthodontics OR orthodontic) AND (CBCT OR (Cone Beam Computed Tomography))	132
EMBASE	‘tooth disease’ AND ‘orthodontics’ AND ‘cone beam computed tomography’ AND [1–1-1966]/sd NOT [30–9-2017]/sd AND [1966–2017]/py	178

#### Data collection and analysis

##### Selection of studies

The studies were screened, selected, and evaluated by two independent authors. Titles and abstracts were examined, and duplicate studies were eliminated. Full texts were obtained when the abstracts did not provide sufficient information. In the second phase of selection, eligibility criteria were used on the full articles. Any discrepancies in the inclusion of articles between reviewers were addressed via discussion until consensus was reached. Disagreements were resolved via discussion and consultation with a third author.

##### Data extraction and management

Two independent authors (Deng and Sun) abstracted study data and evaluated data quality. Disagreements were adjudicated via consensus with a third reviewer (Xu). Data included study design (randomization procedure, blinding and assessment endpoints) and patient characteristics (number, age, author, gender, indication, published years, and orthodontic site). When the data could not be culled from the article, we contacted the authors.

#### Methodological quality assessment

Two independent authors (Deng and Sun) assessed the quality of each study included in the meta-analysis using Methodological Index for Non-randomized Studies (MINORS). Evaluations were compared, and any inconsistencies between the review authors were discussed and resolved. For the self-controlled studies, the MINORS scores ranged from 9 to 15 out of a possible score of 16 (Table 3). There were no clear and consistent inclusion criteria for the included studies, but they were identified as moderate scientific evidence considering their prospective properties and the consecutive inclusion of participants.

**Table 3:**
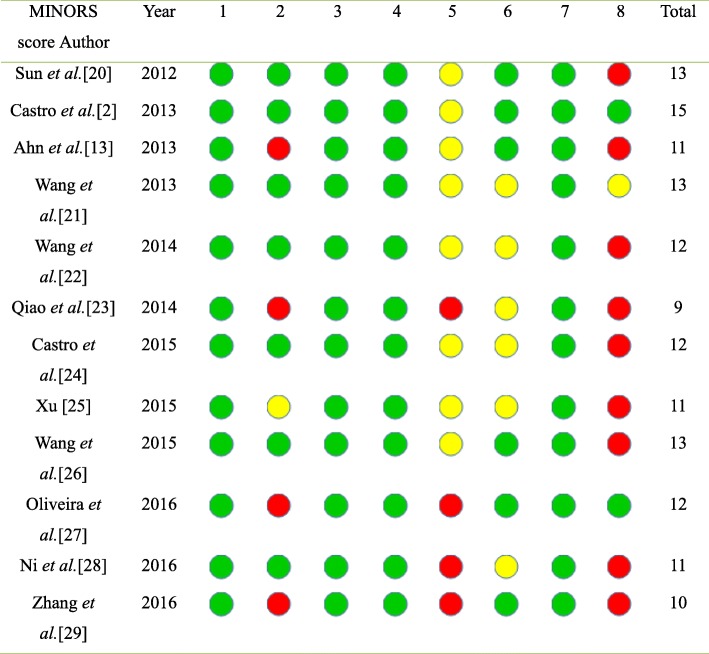
Methodological index for non-randomized studies (MINORS)

Items 1–12 represent: 1, a clearly stated aim; 2, inclusion of consecutive patients; 3, prospective collection of data; 4, endpoints appropriate to the aim of the study; 5, unbiased assessment of the study endpoint; 6, follow-up period appropriate to the aim of the study; and 7, loss to follow-up less than 5%; 8, prospective calculation of the study size. An item scored 0 means not mentioned, 1 means reported but inadequate, and 2 means reported and adequate. The total score was 16 for self-controlled studies. Use red for 0, yellow for 1 yellow and green for 2

#### Statistical analysis

Two authors independently screened the eligible studies, assessed the risk of bias in the trials and extracted data. The following outcomes of interest were recorded: tooth/root length and tooth/root volume. We calculated the mean differences (MD) with 95% confidence intervals (CI) for continuous data and risk ratios (RR) with 95% CI for dichotomous outcomes. Heterogeneity was tested using Cochrane’s Q-test. *I*^*2*^ > 50% was defined to indicate significant heterogeneity (*I*^*2*^-value superior to 25, 50 and 75% corresponding to low, medium and high heterogeneity, respectively). Meta-analyses were performed using Review Manager 5.3 (Nordic Cochrane Centre, Copenhagen, Denmark). If the studies used similar participants and similar interventions, the fixed-effect model was used; if there was potential heterogeneity among studies, we preferred to use random-effects models. If sufficient data were available, we performed the following subgroup analyses: position of tooth; different intervention; extraction group and non-extraction group. If there were insufficient clinical trials for specific interventions or insufficient data for extraction, we qualitatively described the results. We then evaluated the influence of each subgroup on heterogeneity using forest plot analysis. Publication bias was assessed using funnel plot analysis.

## Results

### Description of studies

#### Search results

**Fig. 1:**
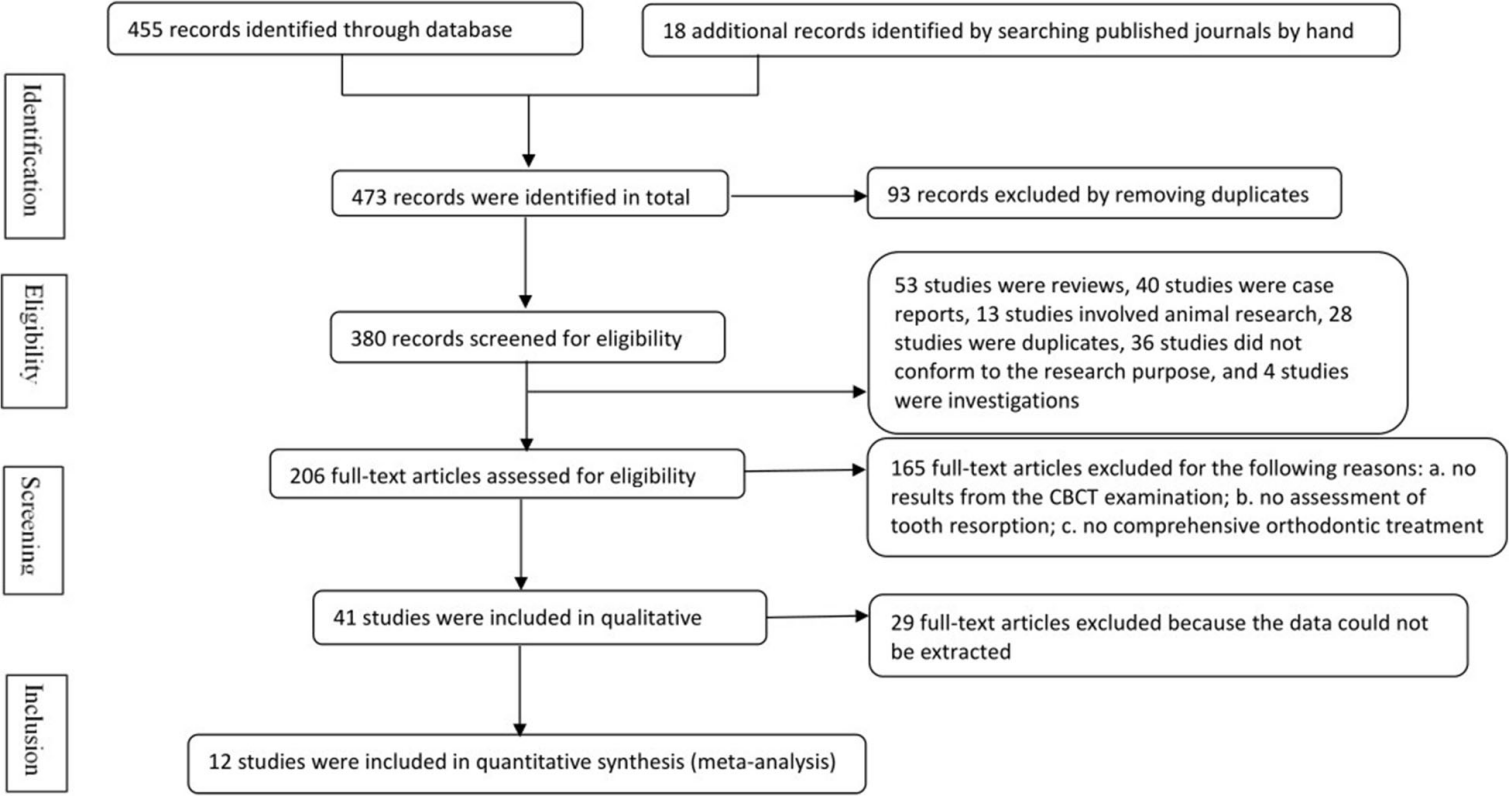
Flowchart of the literature search

A total of 473 studies were obtained from the five databases. All abstracts were entered into the software Endnote (X8). The software screened for duplication, and 380 studies were retrieved. All the remaining studies were screened by the authors. A total of 206 abstracts were retrieved after excluding reviews, case reports, animal research and articles that did not conform to the research purpose. A total of 165 studies were excluded after full-text analysis for the following reasons: a. no results of CBCT examination; b. no assessment of root resorption; and c. local or stage treatment. Among 41 studies, 12 studies were analyzed quantitatively. The search results are presented in Table 2 and the flowchart of the literature search is presented in Fig. 1. Twelve studies were included in this review: Sun et al. [20]; Castro et al. [2]; Ahn et al. [13]; Wang et al. [21]; Wang et al. [22]; Qiao et al. [23]; Castro et al. [24]; Xu [25]; Wang et al. [26]; Oliveira et al. [27]; Ni et al. [28]; Zhang et al. [29].

#### Study characteristics

Study characteristics were summarized in Table 4.

**Table 4:** Characteristics of the included studies

No.	Study		Participants	Outcomes	Evaluated teeth	Indications	Intervention	Duration
1	Sun et al. (2012) [20]		*n* = 17 (M10,F7;23.7y)	TV	(31,32,41,42)	Skeletal Class III	Pre-operative decompensation	7.6 m
2	Castro et al. (2013) [2]		*n* = 30 (M11,F19;13y)	TL	All teeth	Class I malocclusion with crowding	Straight-wire technique (Non-extraction)	22 m
3	Ahn et al. (2013) [13]		*n* = 37 (F37;26.62 ± 8.46y)	RL	Maxillary/Mandibular anterior teeth	Class I dentoalveolar protrusion	Straight-wire technique	1.8 ± 0.4 years
4	Wang et al. (2013) [21]	G1	*n* = 26 (M17,F9; 23.5 ± 3.2)	RL	(31,32,41,42)	Surgical class III	Augmented corticotomy- assisted pre-surgical orthodontics	Unclear
		G2	*n* = 30 (M14,F6; 24.8 ± 3.8)				Conventional procedures	
5	Wang et al. (2014) [22]		*n* = 8 (M5,F3;unclear)	RL	(31,32,41,42)	Severe Class III	Augmented corticotomy- assisted pre-surgical orthodontics	Unclear
6	Qiao et al. (2014) [23]		*n* = 10 (M4,F6;20.6 y)	TL	(11,12,13,21,22,23)	Extraction of 1st premolars	Straight-wire technique	12 m
7	Castro et al. (2015) [24]	G1 G2	*n* = 6 (M2,F4;12.8 ± 1.8y)	TL	Posterior teeth with root-filled; Posterior teeth without root-filled	Permanent dentition Class I malocclusion with moderate dental crowding after RCT	Straight-wire technique	Unclear
8	Xu (2015) [25]		*n* = 18 (M8,F10;25y)	TL	(11,12,13,21,22,23)	Extraction of 1st premolars	Straight-wire technique	Unclear
9	Wang et al. (2015) [26]		*n* = 30 (M13,F17;22.32 ± 2.40y)	TV	(11,12,21,22,31,32, 41,42)	Skeletal Class III	Pre-operative decompensation	9.5 m
10	Oliveira et al. (2016) [27]		*n* = 11 (M5,F6;18-26y)	TL	(11,12,21,22)	Extraction of maxillary first premolars and retraction of maxillary incisors	Edgewise	Unclear
11	Ni et al. (2016) [28]	G1 G2	*n* = 16 (M6,F10; 20.3 ± 3.2y)	TL	(11,12,13,21,22,23)	Moderate crowding in anterior teeth with root-filled; Moderate crowding in anterior teeth without root-filled	Straight-wire technique	18.3 ± 2.6 m
12	Zhang et al. (2016) [29]		*n* = 8 (M3,F5;13.25y)	TL;TV	(12,21)	Anterior cross-bite in earlypermanent dentition	Straight-wire technique	12 m

*TL* tooth length, *RL* root length, *TV* tooth volume, *m* month

##### Characteristics of the participants

A total of 247 participants were investigated and provided 1039 teeth in the 12 studies. The mean age of the participants ranged from 12.8 to 26.62 years, and both genders were included. Three studies [2, 24, 29] were based on teenagers, and the other studies included adults. Participants in four studies [20–22, 26] had skeletal malocclusion that was more serious than other studies, which may be the risk of root resorption.

##### Characteristics of the interventions

All studies included comprehensive orthodontics, not local treatment, and the duration was between pre-treatment and post-treatment. Pre-operative decompensation of orthognathic surgery was also included. However, this type of intervention is different than normal treatment and may be one source of heterogeneity.

##### Characteristics of the outcomes

Tooth length measurement method was primarily based on CBCT data. Tooth length was the distance from the apex to incisal edge or cusp. The volume index measurement method was performed using software for root reconstruction to calculate tooth volume before and after treatment. There was little methodological difference between the studies, and a low measurement bias could be considered.

##### Risk of bias in included studies

The main bias was the implementation bias, which occurred in the process of intervention. Multiple factors of root resorption were also interfering factors. Three articles [2, 20, 26] exhibited report bias.

## Effects of interventions

### Primary results

#### Tooth length—total teeth

**Fig. 2:**
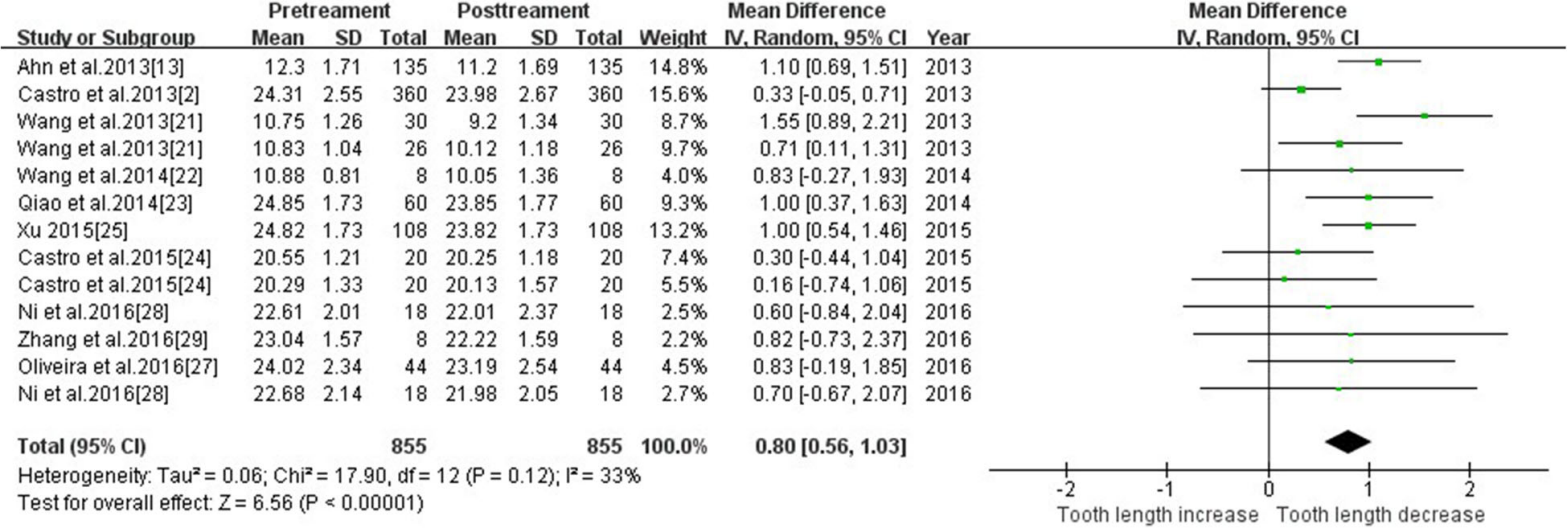
Primary result of tooth length increase or decrease with orthodontic treatment—total teeth

**Fig. 3:**
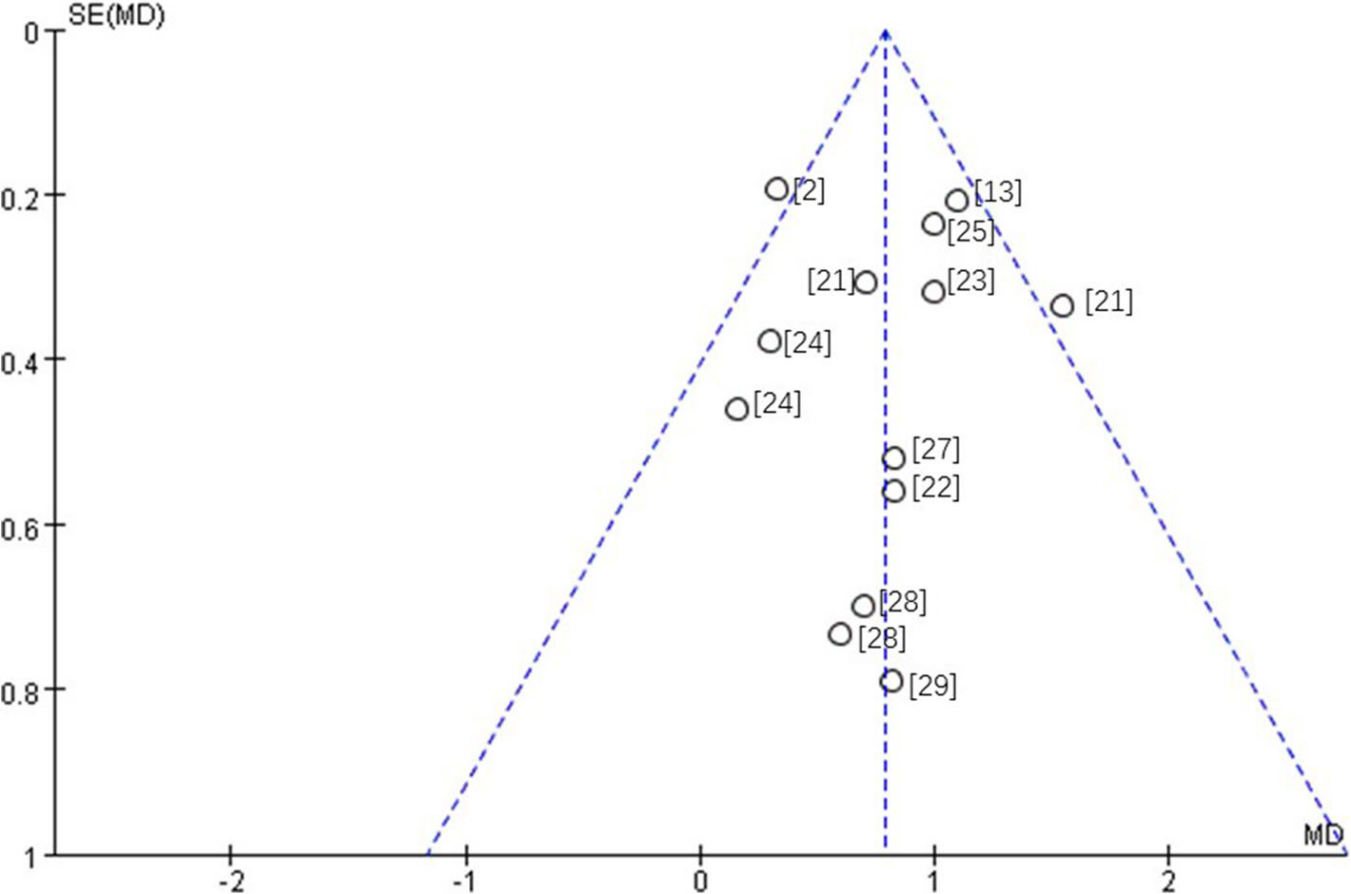
Funnel plot—root resorption in total teeth. Included studies: Castro et al. [2]; Ahn et al. [13]; Wang et al. [21]; Wang et al. [22]; Qiao et al. [23]; Castro et al. [24]; Xu [25]; Oliveira et al. [27]; Ni et al. [28]; Zhang et al. [29]

Ten studies reported changes in tooth length using CBCT. Meta-analysis revealed medium heterogeneity (I^2^ = 33%), and a random effect model was used. Post-treatment was comparable to pre-treatment (MD = 0.80, 95% CI 0.56, 1.03, *P* < 0.00001). The meta-analysis results are shown in Fig. 2. The funnel plot was more symmetrical, which indicates a small publication bias (Fig. 3). The source of heterogeneity may lie in the method of measurement. Ahn et al. and Wang et al.’s studies [13, 21, 22] measured the root length, which was the distance from the apex to the enamel dentin boundary, but other studies measured the tooth length as the distance from the cusp or incisal edge to apex.

#### Tooth length—maxillary central incisor

**Fig. 4:**
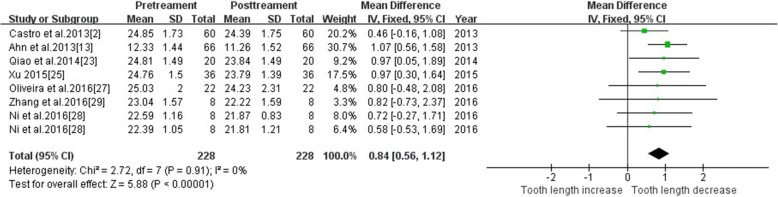
Primary result of tooth length increase or decrease with orthodontic treatment—maxillary central incisor

Seven studies reported changes of tooth length in maxillary central incisors using CBCT. Tooth length was significantly reduced after orthodontic treatment (MD = 0.84, 95% CI 0.56, 1.12, *P* < 0.00001). The meta-analysis results are shown in Fig. 4.

#### Tooth length—maxillary lateral incisor

**Fig. 5:**
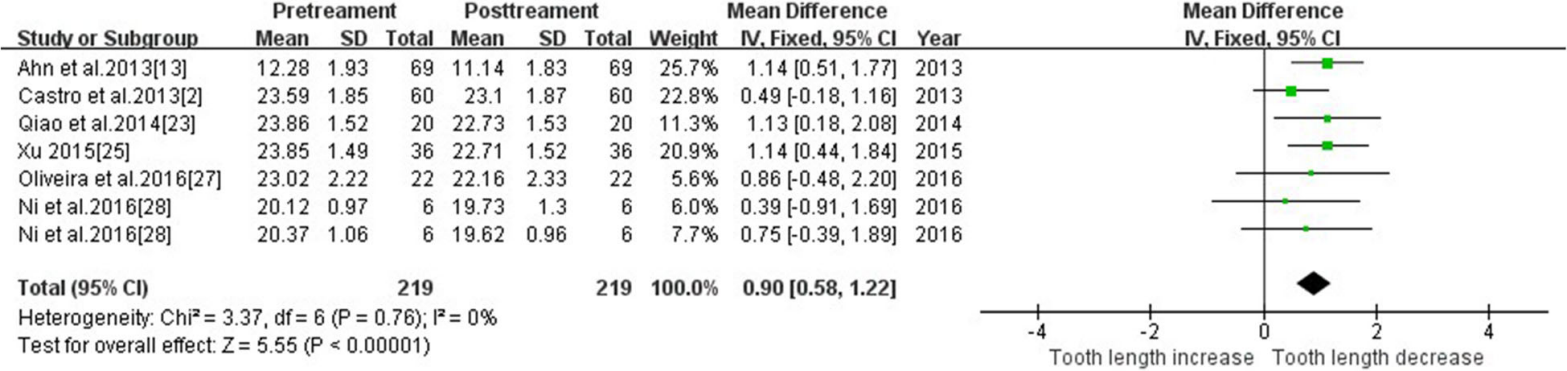
Primary result of tooth length increase or decrease with orthodontic treatment—maxillary lateral incisor

Six studies reported changes of tooth length in maxillary lateral incisors using CBCT. Tooth length of maxillary lateral incisors was significantly reduced after treatment (MD = 0.90, 95% CI 0.58, 1.22, *P* < 0.00001). The meta-analysis results are shown in Fig. 5.

#### Tooth length—maxillary canine

**Fig. 6:**
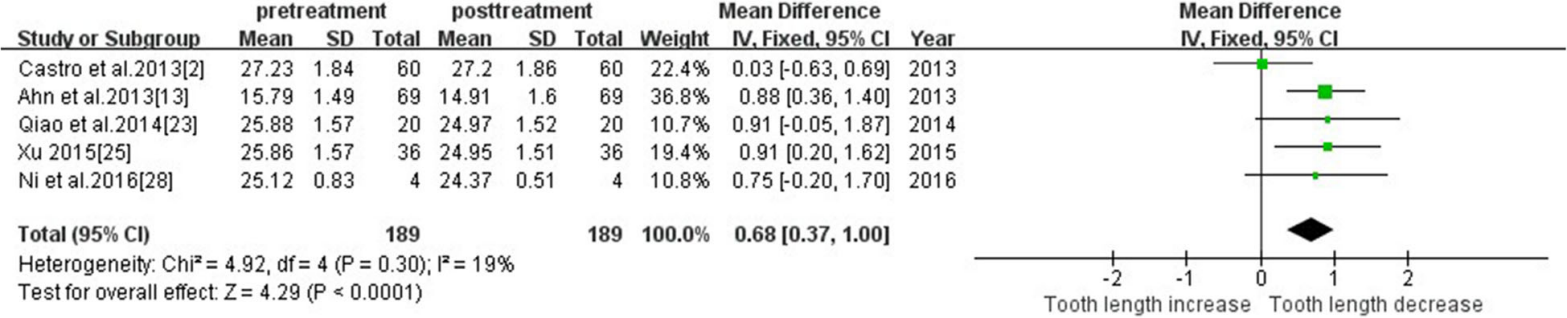
Primary result of tooth length increase or decrease with orthodontic treatment—maxillary canine

Five studies reported changes in maxillary canine. Meta-analysis revealed low heterogeneity (I^2^ = 19%), and a fixed-effect model was used. The tooth length was significantly shorter after treatment (MD = 0.68, 95% CI 0.37, 1.00, *P* < 0.00001). The meta-analysis results are shown in Fig. 6.

#### Tooth length— mandibular anterior teeth

**Fig. 7:**
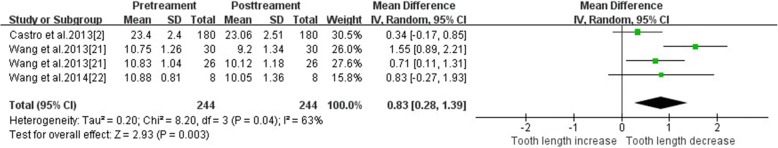
Primary result of tooth length increase or decrease with orthodontic treatment—mandibular anterior teeth

**Fig. 8:**
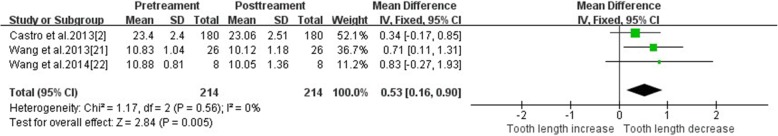
Primary result of tooth length increase or decrease with orthodontic treatment—mandibular anterior teeth after removing control group of Wang et al. [21]

Three studies reported changes of tooth length in mandibular anterior teeth using CBCT. Meta-analysis revealed high heterogeneity (I^2^ = 63%), and a random-effect model was used. Sensitivity tests were performed after the removal of different study groups in Wang et al. [21], and the heterogeneity was reduced to 0%. Post-treatment was comparable to pre-treatment (MD = 0.53 95% CI 0.16, 0.90, *P* < 0.00001). The meta-analysis results are shown in Figs. 7 and 8.

#### Tooth length—tooth extraction & non-tooth extraction

**Fig. 9:**
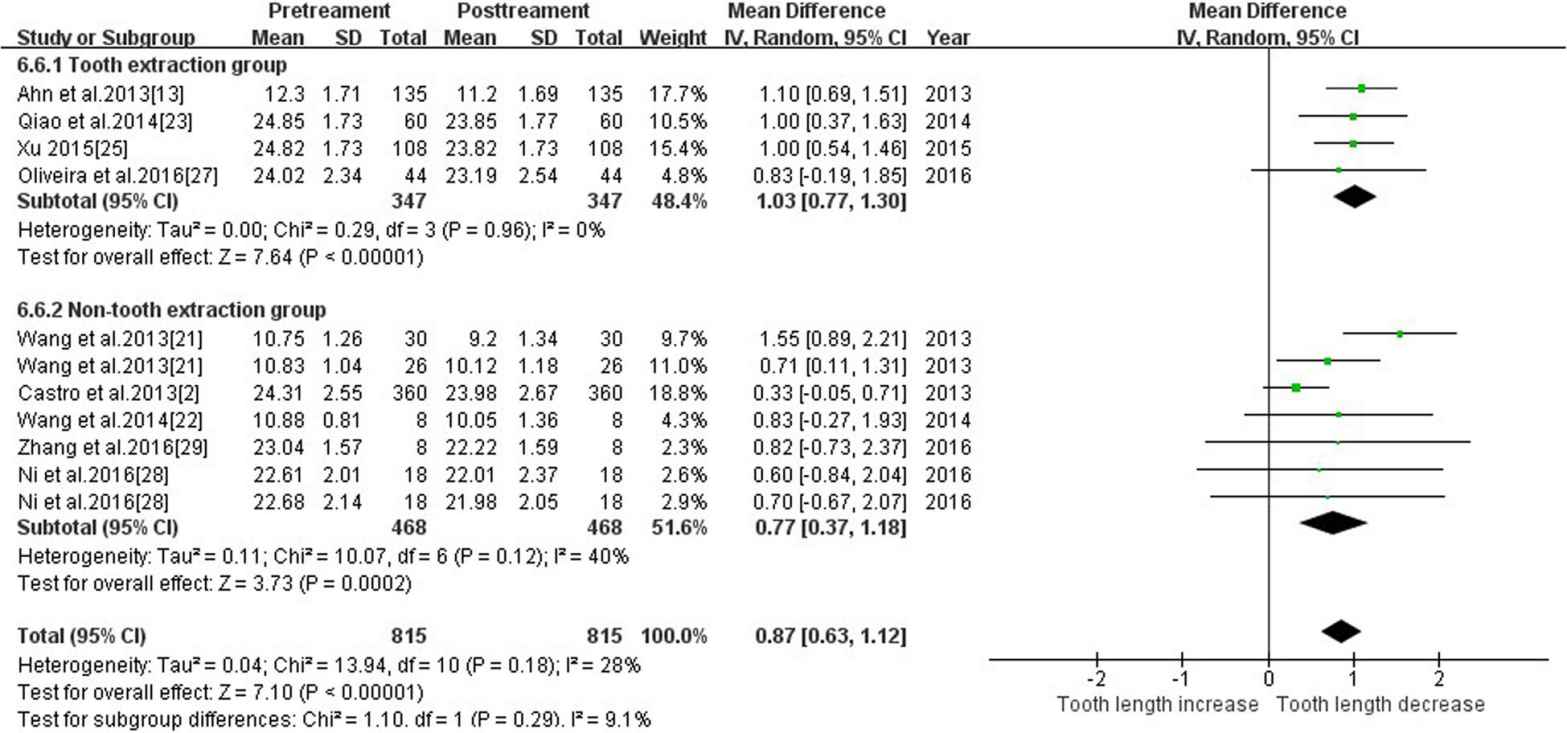
Primary result of tooth length increase or decrease with orthodontic treatment—extraction or not

The study was divided into two subgroups, tooth extraction and non-extraction, based on the orthodontic approach. The following heterogeneity test results were demonstrated: *p* = 0.96 and 0.12, I^2^ was 0 and 40%, respectively; the two subgroups combined together *p* = 0.18, I^2^ was 28%. Subgroup analysis demonstrated CHI^2^ = 1.10, *P* = 0.29, and subgroup differences were not statistically significant. Therefore, we speculated whether extraction or not was less affected to heterogeneity. The heterogeneity source in the non-extraction group arose from control group in Wang et al. [21]. The overall effect value (Z = 7.10, *P* < 0.00001) suggests that the effect of extraction treatment on tooth length was statistically significant. The total effect of the tooth extraction group was 1.03 [0.77, 1.30], and the total effect value of the non-extractive group was 0.77[0.37, 1.18]. Tooth extraction may have caused more root resorption. The meta-analysis results are shown in Fig. 9.

#### Tooth length—different interventions

**Fig. 10:**
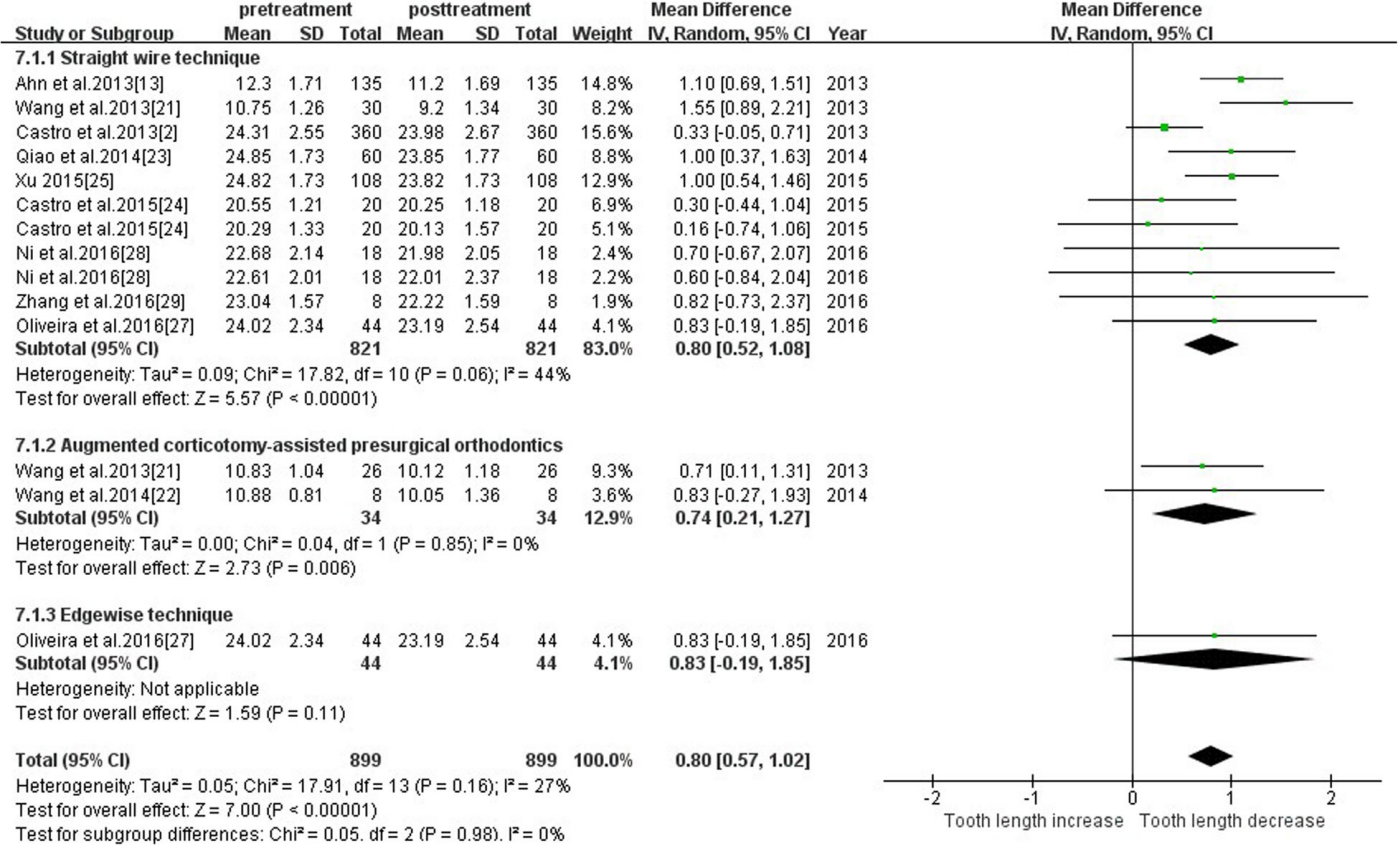
Primary result of tooth length increase or decrease with orthodontic treatment—different orthodontic technique

The study was divided into three subgroups, straight wire, augmented corticotomy-assisted presurgical orthodontics and edgewise technique, based on the orthodontic technique. Subgroup analysis demonstrated that subgroup differences were not statistically significant. Therefore, we speculated different intervention of heterogeneity was less affected. The total effect of the straight wire group was 0.8 [0.52, 1.08], the total effect value of the augmented corticotomy-assisted presurgical orthodontics group was 0.74[0.21, 1.27] and the total effect of the edgewise group was 0.83[− 0.19, 1.85]. The augmented corticotomy-assisted presurgical orthodontics may cause less root resorption. The meta-analysis results are shown in Fig. 10.

#### Root volume

**Fig. 11:**
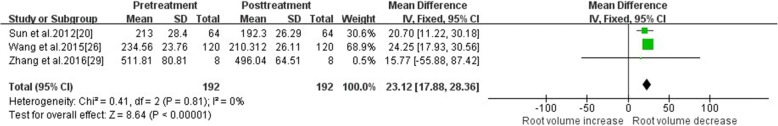
Primary result of root volume increase or decrease with orthodontic treatment

Three studies reported changes in root volume using CBCT. The results of meta-analysis revealed that the root volume before and after orthodontic treatment was significantly different (MD = 23.12, 95% CI 17.88, 28.36 *P* < 0.00001). The root volume was significantly reduced after treatment. The meta-analysis results are shown in Fig. 11.

## Discussion

### Summary of main results

Evidence suggests that orthodontic treatment causes an increased incidence and severity of apical root resorption. Tooth length and root volume were reduced after orthodontic intervention.

### Overall completeness and applicability of evidence

Numerous CBCT studies investigated root resorption before and after orthodontic treatment, but quantitative data of the extraction were only available in 12 articles for a meta-analysis. We chose two major indicators, tooth/root length and root volume, to reflect root resorption. Other indicators that reflect changes were not included, and numerical integration and meta-analysis were not performed. Quantitative analysis of other indicators may be performed using numerical analyses to improve the data on this issue.

### Quality of the evidence

Twelve articles were included in this meta-analysis, and all of the studies were non-randomized controlled trials. Randomized controls cannot be used because it is ethically impossible to perform CBCT screenings in patients who have not undergone orthodontic treatment. All studies were self-controlled, which reflects the impact of intervention on these patients more accurately. All patients were diagnosed and measured using CBCT, which excluded the shortcomings of magnification and distortion in 2D image data. All data sources were evaluated more accurately and reproducibly by CBCT, which is the most accurate method of obtaining data in vivo. Methodological evaluation scores from 9 to 15, and 4 studies received scores that were above or equal to 13, which were considered as high quality. One study was below 10 and was considered low quality. The remaining studies were of medium quality. The overall quality of the selected literature was good. There were 3 prospective trials, and the remaining trials were retrospective experiments. Prospective experimental evidence will provide more adequate data.

### Potential biases in the review process

The different orthodontic technologies, such as straight wire and edge wise, and corticotomy, incompleteness of some of the reports and lack of quality control in some trials may have contributed to bias in study assessments. We made every attempt to limit bias in the review process by ensuring a comprehensive search for potentially eligible studies. Time limitations prevented the search of additional databases and sources, which may have identified additional published and unpublished studies. There may also be publication bias because of the lack of publication of negative results. We strictly controlled the inclusion of exclusion criteria. There were only two prospective experiments, while others were retrospective studies. There may be biases in sample selection and dropout. The sample size of all studies was relatively small, and only one of the studies calculated the sample size [27].

The age distribution did not completely cover the age of patients with orthodontic treatment, but it covered the span of treatment for most patients. However, there were few samples for adolescent studies, and many patients of orthodontic treatment are adolescents in clinical practice. Therefore, whether the conclusions of this meta-analysis are well suited for adolescent samples must be further studied.

#### Primary outcome tooth length

##### Tooth position

Total tooth root length was reduced after orthodontic treatment, and the included studies exhibited a small publication bias. The effect value indicated that the maxillary lateral incisors were the most absorbed, followed by the maxillary central incisors, mandibular anterior teeth and upper canines. Nanekrungsan et al. [8] found that the maxillary lateral incisors were primarily reduced after treatment. Yu et al. [30] found that the maxillary lateral incisors exhibited greater resorption than maxillary central incisors and canines using CBCT. Kennedy et al. [31] also found that the maxillary lateral incisors were more prone to root resorption than the central incisors. Pejicic et al. [32] found that lateral incisors were primarily affected, and mean values ranged from 0.5 mm to 3 mm, which is consistent with the conclusions of our study. Previous 2D studies [3, 33, 34] found that maxillary central incisors were the most affected teeth. Sameshima et al. [11] demonstrated that the absorption order was the upper central incisors, the upper lateral incisors, the lower central incisors, and the lower lateral incisors. Jung et al. [35] found that the maxillary central incisors were the most resorbed, with 27% undergoing greater than 1 mm of root resorption. Inaccuracies caused by the magnification and overlap of 2D data, the different types of patient malocclusion, or the different treatment methods may explain the differences in absorption of the anterior teeth.

A meta-analysis of Segal et al. [36] was based on 2D data and demonstrated a strong correlation between root resorption and apical displacement. The mean resorption of upper central incisors was 1.421 ± 0.448 mm, which was slightly higher than the present study (MD = 0.84,CI[0.56,1.12]). Two-dimensional data may over-estimated root resorption.

The present meta-analysis revealed that the root resorption of the upper central incisors and upper lateral incisors were similar, and it was difficult to determine which tooth was the most affected. The sample of 3D studies was quite small. Therefore, larger 3D sample sizes and more clinical trial evidence are required to supplement and confirm these conclusions.

The heterogeneity of root resorption of the mandibular anterior teeth was high partially because two studies measured the root length and other studies measured tooth length. Removal of the data from control group of Wang et al. [21] reduced the heterogeneity to zero, which suggested that this data was the source of heterogeneity. This group was pre-operative orthodontic of orthognathic surgery patients, which resulted in greater root resorption (1.55 ± 0.66 mm). Experimental group was also pre-operatively compensated, but corticotomy was added. A previous study demonstrated that corticotomy reduced the duration of treatment and resulted in lower root resorption compared to traditional methods. Therefore, the value of experimental group may be reduced.

The key of orthodontics prior to orthognathic surgery lies in the anterior teeth for compensation [37]. The root much more easily touches the alveolar bone, which increases the risk of root resorption [38]. Some scholars confirmed that maxillary root resorption is most likely to occur in the patients with bony malocclusion because of the maximum distance of tooth movement, especially the incisors [39, 40]. Therefore, the data suggest that orthopedic orthodontic treatment of skeletal malocclusion using simple orthodontics will cause more root resorption, regardless of cortical osteotomy intervention.

##### Extraction vs non-extraction

There is controversy about root resorption following extraction and non-extraction methods. The present study was divided into two subgroups: extraction group and non-extraction group. The heterogeneity test found no significant difference between the subgroups. Therefore, the effect of extraction on the heterogeneity is speculative. The overall effect value suggests that the effect of extraction treatment on tooth length was statistically significant. The heterogeneity source of the non-extraction group arose from control group in Wang et al. [21], whose analysis was based on tooth position.

The effect of tooth extraction (1.03 ± 0.27) on root resorption was greater than that in the non-extraction group (0.77 ± 0.40). Baumrind et al. [41] found no association between tooth extraction and root resorption in a 2D study, which is consistent with the Kaley et al. [42] study. However, Sameshima et al. [11] found that removal of four first premolar teeth resulted in more root resorption than removal of two maxillary premolar teeth or no removal. The incidence of EARR was 3.72 times higher in patients who received extractions than patients without extraction [43]. Sun et al. [44] further confirmed that tooth extraction compared to non-extraction treatment caused more root resorption, which is consistent with the conclusions of the present meta-analysis. Jung et al. [35] found that maxillary central incisors resorption was 0.6 ± 0.67 mm in the non-extraction group and 0.98 ± 0.82 mm in the extraction group. The mean values are close to the present meta-analysis, but the standard deviation was smaller in our meta-analysis than in his study. In conclusion, the extraction group exhibited greater RR than the non-extraction group. The three-dimensional data were more accurate, and the root resorption that occurred in clinical treatment was not as serious as expected.

##### Different interventions

According to the subgroup analysis of the intervention methods, the intervention was not considered as the main source of heterogeneity in this study. According to the effect value from largest to smallest of root resorption was edgewise, straight wire and augmented corticotomy-assisted presurgical orthodontics. However, there was only one study of edgewise, so more clinical studies were needed for the comparison between edgewise and other orthodontic techniques. The corticotomy group had a slightly lower root resorption than the straight wire group, suggesting that corticotomy may also reduce tooth root resorption in addition to accelerating tooth movement [45].

#### Primary outcome tooth volume

Root resorption is not just a two-dimensional change in length, it actually occurs in three dimensions, including on the buccal-lingual and mesial-distal sides. Therefore, the volume index is more realistic than the length index, which reflects the root resorption accurately. All three studies [20, 26, 29] focused on the maxillary and mandibular incisors and were consistent with no heterogeneity in the length index.

#### Factors affecting root resorption

##### Treatment duration

Clinical studies demonstrated that a long course of treatment may cause more serious root resorption [41, 46–48]. Therefore, clinical orthodontists should avoid prolonged orthodontic treatment. However, if the patient has had root resorption or root resorption prior to treatment, orthodontic treatment will increase the degree and speed of root resorption, which causes severe root resorption [46]. However, some studies reported no correlation between treatment duration and apical root loss [34, 49].One possible reason is that patients prolonged the treatment because of untimely referral. The limited periods of activation during the prolonged referral period in a lighter force system may also explain this difference. Harry et al. [50] suggested that the stress duration is the more serious reason than the magnitude of force. Segal et al.’s meta-analysis based on 2D data demonstrated a strong correlation between root resorption and duration [36].

Treatment duration is a risk factor of root resorption, but it is also correlated with it. We included data before and after comprehensive orthodontic treatment rather than topical or stage treatment to exclude changes in tooth length that occurred only after months of adduction or depression. Normal orthodontic treatment of a general course is 2–3 years, and stage data may not reflect the complete response to an entire treatment. The present study included before and after treatment data, which reduced the bias to some extent. These results provide the best available evidence for clinical decisions to minimize the risk and severity of apical root resorption. Therefore, clinicians should be very careful when moving anterior teeth over a long distance and a long time.

## Limitations

1. There were many confounding factors, such as tooth movement distance, magnitude of force, alveolar shape and technique. There was no regression analysis of age, gender or other risk factors. Original studies did not meet consistent inclusion and exclusion criteria and provided limited information about age and could not be compared quantitatively. We hope that more unified reference planes and measurement methods may be used in future quantitative analyses.
2. Only non-randomized controlled trials (self-controlled trials) were included because of the lack of a large number of related RCT experiments due to limited conditions.
3. Most of the included trials (9 papers) resulted in changes in the length of the tooth, and root resorption was a three-dimensional change, not only in two dimensions. However, only three studies included volume changes.
4. Most of the research focused on the upper and lower anterior teeth, and root resorption of the upper and lower posterior teeth was less studied. Therefore, total dentition root resorption severity and impact factors require further experiments to verify.

## Clinical significance

It is necessary to inform patients of the risk of root resorption prior to orthodontic treatment. Significant shortening of the root length will lead to inappropriate crown-root ratios and adjacency to the periodontal tissue. Apical resorption greater than 3 mm is equivalent to 1 mm of bone loss, which will accelerate the periodontal disease process. The treatment program must be re-evaluated if serious root resorption is found. Future experiments should be based on a reasonable sample size and the implementation of the entire orthodontic process to ensure the effect value.

The above meta-analysis demonstrated that the average value of root resorption was approximately 1 mm, and the upper limit did not exceed 2 mm. Sharpe’s grading standards indicated that the first grade was slightly blunt (1–2 mm) [51]. The results were grade 1. Malmgren’s rating standard indicates that level 1 is irregular root contour and level 2 is root resorption apically amounting to less than 2 mm (minor resorption) [40]. The most serious case did not exceed 2 degrees, and it was a mild absorption.

## Conclusions

The following conclusions are drawn based on the existing research:

1. Evidence suggests that orthodontic treatment increased the incidence and severity of apical root resorption. Tooth length and root volume were reduced after orthodontic intervention, but these changes were in a clinically acceptable range.
2. Different tooth positions exhibited different degrees of absorption, and the sequence of RR from heaviest to lightest was maxillary lateral incisors, maxillary central incisors, mandibular anterior teeth, and maxillary canines.
3. Tooth extraction may result in more root resorption than non-extraction.
4. Most of the patients measured using CBCT exhibited root resorption within the clinically acceptable range. The RR value of CBCT was lower than the 2D data.

However, more methods are needed to provide more reliable evidence for clinical trials based on CBCT data. Experimental studies on treatment, the distance and angle of tooth movement, and correction techniques may provide more clinical guidance for orthodontic treatment of RR reduction.

## Abbreviations

2D: Two-dimensional
3D: Three-dimensional
CBCT: Cone beam computed tomography
CNKI: China National Knowledge Infrastructure
EARR: External apical root resorption
m: Month
MINORS: Methodological index for non-randomized studies
PRISMA: Preferred Reporting Items for Systematic Reviews and Meta Analyses
RL: Root length
RR: Root resorption
TL: Tooth length
TV: Tooth volume
